# Poly(β-amino ester)s-Based Delivery Systems for Targeted Transdermal Vaccination

**DOI:** 10.3390/pharmaceutics15041262

**Published:** 2023-04-17

**Authors:** Núria Puigmal, Víctor Ramos, Natalie Artzi, Salvador Borrós

**Affiliations:** 1 Grup d’Enginyeria de Materials (GEMAT), Institut Químic de Sarrià, Universitat Ramon Llull, 08017 Barcelona, Spain; nuriap@mit.edu (N.P.);; 2 Department of Medicine, Division of Engineering in Medicine, Brigham and Women’s Hospital, Harvard Medical School, Boston, MA 02115, USA; nartzi@bwh.harvard.edu; 3 Institute for Medical Engineering and Science (IMES), Massachusetts Institute of Technology, Cambridge, MA 02139, USA; 4 Wyss Institute for Biologically Inspired Engineering, Harvard University, Boston, MA 02115, USA

**Keywords:** poly(β-amino ester)s, targeted delivery, mannose, transdermal vaccination, plasmid DNA

## Abstract

Nucleic acid vaccines have become a transformative technology to fight emerging infectious diseases and cancer. Delivery of such via the transdermal route could boost their efficacy given the complex immune cell reservoir present in the skin that is capable of engendering robust immune responses. We have generated a novel library of vectors derived from poly(β-amino ester)s (PBAEs) including oligopeptide-termini and a natural ligand, mannose, for targeted transfection of antigen presenting cells (APCs) such as Langerhans cells and macrophages in the dermal milieu. Our results reaffirmed terminal decoration of PBAEs with oligopeptide chains as a powerful tool to induce cell-specific transfection, identifying an outstanding candidate with a ten-fold increased transfection efficiency over commercial controls in vitro. The inclusion of mannose in the PBAE backbone rendered an additive effect and increased transfection levels, achieving superior gene expression in human monocyte-derived dendritic cells and other accessory antigen presenting cells. Moreover, top performing candidates were capable of mediating surface gene transfer when deposited as polyelectrolyte films onto transdermal devices such as microneedles, offering alternatives to conventional hypodermic administration. We predict that the use of highly efficient delivery vectors derived from PBAEs could advance clinical translation of nucleic acid vaccination over protein- and peptide-based strategies.

## 1. Introduction

Delivery of nucleic acid therapeutics is now at the forefront of global efforts to combat a plethora of conditions [1], from emerging infectious diseases to cancers, catalyzed by the full approval of mRNA-based vaccines against COVID-19 [2,3]. Nucleic acid vaccination offers an attractive alternative to traditional approaches which entail major drawbacks such as complex manufacturing, limited cell-mediated immunity, and safety concerns due to potential genomic integration or morphing into pathogenic forms [4]. Contrastingly, nucleic acid vaccines offer enhanced biocompatibility, cost-effective production [5], and the capacity to engender both cellular and humoral immune responses [6], providing a paradigm for rapid vaccine development and pandemic preparedness. Building on this momentum, routes of vaccination other than the intramuscular are now being explored to increase their potency and facilitate global implementation. The skin, being an immune sentry-rich tissue, has been proposed as a superior route for immunization over the intramuscular since it hosts a profoundly richer network of antigen-presenting cells (APCs) contributing to the immunocompetence of the skin [7,8]. However, the first-generation of transdermal vaccines proved to be poorly immunogenic in humans due to inefficient gene delivery and weak cell transfection [9], evidencing the need for more refined delivery vectors to enhance immunization levels [10,11]. Poly(β-amino ester)s (PBAEs) are highly regarded delivery vectors due to their excellent biocompatibility, biodegradability, and an ease of synthesis without solvents nor catalysts [12,13,14]. Given their chemical flexibility, extensive libraries of PBAEs can be generated by combinatorial end-modification to fine-tune their physicochemical properties such as size or zeta potential and promote cell-specific delivery of nucleic acids [15]. Our group has long reported end-modification of PBAEs with amine-rich oligopeptides as a powerful tool to drastically boost transfection efficiencies in a cell-type-specific manner while also improving endosomal escape and biocompatibility [16,17]. In the context of transdermal gene vaccination, targeting professional APCs such as Langerhans cells (LCs) has shown great therapeutic promise as they are key instigators of immune responses in the dermal milieu [18,19]. In this work, a new library of mannose-modified (MM)-PBAEs combined with oligopeptide-modified (OM)-PBAEs was developed for targeted delivery to APCs. Specifically, we leveraged the mannose-binding affinity of the Langerin receptor and the mannose receptor—expressed by LCs [20] and macrophages, respectively [21]—to generate PBAE candidates promoting APC transfection by virtue of their mannose-binding capacity. Beyond polyplexes, less-known PBAE-derived systems such as polyelectrolyte films (PEMs), gels, or fibers might be more convenient for clinical implementation [22,23] since their solvent-free nature and extended shelf-life could allow widespread distribution to developing countries [24]. Polyelectrolyte films (PEMs) can virtually cover any substrate, allowing surface-mediated transfection through the skin when deposited onto transdermal delivery devices [24,25,26]. Here, we also studied the transfection potential of OM- and MM-PBAEs when deposited as PEMs onto microneedles (MNs) as they offer self-administered, needle-free, and painless means for transdermal vaccination when integrated with PBAE-based systems [27,28,29,30,31]. We report that active targeting with mannose, if helpful, does not dictate transfection efficacy as it is the inclusion of oligopeptides that ultimately rendered cell-specific targeting in APCs. We also proved the flexibility of PBAEs as delivery systems when coupled to transdermal devices such as MNs, opening up their potential use as solvent-free vectors for both prophylactic and therapeutic avenues.

## 2. Materials and Methods

### 2.1. Materials

Reagents and solvents used for polymer synthesis and fabrication of transdermal devices were purchased from Sigma-Aldrich (St. Louis, MO, USA) and Panreac (Barcelona, Spain) unless stated otherwise. Oligopeptide moieties used for polymer decoration (H-Cys-Arg-Arg-Arg-NH2, H-Cys-Lys-Lys-Lys-NH2, H-Cys-His-His-His-NH2, and H-Cys-Asp-Asp-Asp-NH2) were obtained from GL Biochem Ltd. (Shanghai, China) with a purity higher than 98%. Cell lines were obtained from ATCC (Manassas, VA, USA). Human monocyte-derived dendritic cells obtained from healthy donors were kindly provided by Dr. Francesc Català-Moll (IDIBELL, Barcelona, Spain). Plasmid reporter green fluorescent protein (pmaxGFP, 3486 bp) was purified using the NucleoBond^®^ Xtra Midi Plus EF kit (Macherey-Nagel, Dueren, Germany) from competent *Escherichia coli* cells.

### 2.2. Animals

Adult 6–8 weeks old C57BL/6 mice were purchased (Envigo, Indianapolis, IN, USA) and kept under pathogen-free conditions in laminar flow boxes. Animal maintenance and experiments were performed in accordance with established guidelines of the Catalan Government and following protocol number 8856, approved by the Direcció General del Medi Natural.

### 2.3. Synthesis of Oligopeptide-Modified PBAE Polymers

Oligopeptide-terminated PBAEs were synthesized as described by Dosta and colleagues [32]. Briefly, end-modified PBAE polymers were obtained by Michael addition via a two-step synthetic strategy. First, polymerization of the C6 acrylate-terminated polymer was performed using 5-amino-1-pentanol (0.426 g, 4.1 mmol), hexylamine (0.422 g, 4.1 mmol), and 1,4- butanediol diacrylate (2.0 g, 9.1 mmol) reacted at 90 °C for 24 h. Secondly, amine-moieties were end-capped with oligopeptides (1:2.5 M ratio of acrylate:oligopeptides) in dimethyl sulfoxide (DMSO). The mixture was stirred overnight at room temperature and the resulting polymer was precipitated with a mixture of diethyl ether and acetone (70:30 *v*/*v*). Oligopeptides used for the end-capping reaction were: Cys + 3Arg (CR3), Cys + 3Lys (CK3), Cys + 3His (CH3), and Cys + 3Asp (CD3). Synthesized structures were confirmed by ^1^H NMR, recorded in a 400 MHz Varian (NMR Instruments, Claredon Hills, IL, USA) in methanol-d4.

### 2.4. Synthesis of Mannose-Modified PBAE Polymers

Synthesis of a second generation of oligopeptide-modified PBAEs including mannose moieties was performed as follows. Stock solutions of tri-arginine and tri-lysine end-modified PBAEs (CR3-C6, CK3-C6) (100 mg mL^−1^) were dissolved in anhydrous DMSO with allyl-α-D-Mannopyranoside (ADM) and triethylamine (1:4:10 M ratio). Reactions were allowed to proceed overnight at room temperature under vigorous stirring and resulting polymers were precipitated with a mixture of diethyl ether and acetone (70:30 *v*/*v*).

### 2.5. Formation and Physicochemical Characterization of PBAE-Derived Polyplexes

Complexes containing pDNA (encoding for the green fluorescence protein) were obtained as described by us previously [16] by mixing equal volumes of nucleic acid solution (0.5 mg mL^−1^) and PBAE stock solution (100 mg mL^−1^ in DMSO) to achieve the desired polymer-to-nucleic acid ratio (*w*/*w*). In brief, the pDNA solution was added over the polymer solution, both diluted in sodium acetate buffer (AcONa buffer; 12.5 mM, pH = 5), mixed by vigorous pipetting and allowed to react for 15 min at room temperature. Combinations of polymers used for nanoparticle formation were prepared as follows. Positively charged OM-PBAEs (CR3-, CK3-, CH3-C6, and their mannosylated counterparts) were mixed in a 50:50 ratio, whereas pairs including both cationic and anionic polymers (CR3-, CK3-, CH3-, Man-CR3-, or Man-CK3-C6 with CD3-C6) presented a 70:30 ratio.

Resulting nanoparticles were characterized by agarose gel electrophoresis and dynamic light scattering. Nucleic acid retardation was assessed by loading PBAE-derived polyplexes into the wells of agarose gels (0.8% *w*/*v*) using different polymer-to-nucleic acid ratios. Particle-size distribution and zeta distribution of resulting polyplexes was evaluated by dynamic light scattering (DLS) in a Nanosizer ZS Instrument (Malvern Instruments, Malvern, UK) using different polymerto-pDNA weight ratios ranging from 5:1 to 150:1.

### 2.6. Fabrication of Polymeric Rods

Polymeric rods mimicking the features of subcutaneous contraceptive delivery systems were fabricated using a stainless-steel rotary extruder. Polycaprolactone (PCL) pellets were charged in the loading container of the extruder and melted at 80 °C for 10 min. Next, the instrument was allowed to cool down for safe manipulation and PCL strands were extruded and cut rendering 1 cm-rods with 1 mm diameter.

### 2.7. Fabrication of Solid and Dissolvable Microneedles

Stainless steel microneedles sizing 500 µm and used for cosmetic purposes served as positive molds. Female molds were fabricated by pouring a PDMS solution (Sylgard^®^ 184) prepared as recommended by the manufacturer over the positive mold and allowing it to cure for 24 h at room temperature. To fabricate solid PLGA-based microneedles, a 15% *w*/*v* solution of PLGA (Resomer^®^ RG 858 S, Sigma-Aldrich) was dissolved in acetonitrile, degasified for 30 min, and casted on top of the molds. Solid MNs were allowed to dry under vacuum for 24 h and stored at room temperature. Dissolvable microneedles were fabricated in a similar fashion. Briefly, a premixed solution of poly(vinyl alcohol) (Mw 89,000–98,000) and glycerol (10% and 2% *w*/*v,* respectively, in deionized water) was combined with a suspension of PBAE-derived polyplexes and poured on the mold. Microneedles were allowed to dry likewise and stored until further use.

### 2.8. Layer-by-Layer Deposition of PBAEs and Characterization on Transcutaneous Delivery Devices

Manual deposition of polyelectrolyte multilayers of PBAE:pDNA on various substrates was adapted from elsewhere [33]. Substrates including extruded rods and solid polymeric MNs were immersed in a PBAE solution (2 mg mL^−1^) in either AcONa buffer or PBS) for 5 min followed by two successive washing steps of 30 s each using the same buffer. Next, substrates were submerged in the pDNA solution (1 mg mL^−1^) during 5 min and washed in the same fashion. This procedure was repeated multiple times until achieving the desired number of PBAE:pDNA bilayers, and stored at room temperature until use. Characterization of in situ polyplex formation was assessed by DLS. Polymeric rods precoated with polyelectrolyte films were submerged in PBS for 30 min and supernatants were collected for DLS analysis. Quantification of deposited DNA was conducted using the Quant-iT™ dsDNA Assay Kit High Sensitivity (Thermo Fisher Scientific, Spain) following the manufacturer’s instructions. Extruded rods were coated as described and placed in Eppendorf tubes containing 50 µL of PBS to allow film release for 30 min. Supernatant was next collected and analyzed by fluorescence (λ = 485/530) using a plate reader (Elx808 Biotek Instruments Ltd., Winooski, VT, USA).

### 2.9. Ex Vivo Skin Penetration and Film Deposition Studies

The ability of transdermal devices to penetrate the skin was tested ex vivo using 2 × 2 cm skin explants harvested from healthy C57BL/6 mice. MNs were thumb-pressed for 10 min; whereas rods were implanted using an 18-gauze syringe simulating clinical guidelines. Transdermal skin penetration was confirmed by surface staining with Trypan blue (0.4%) and further imaged by optical microscopy. Film deposition following transdermal delivery was assessed by fluorescence microscopy. Briefly, devices were coated with polyelectrolyte films as described before using a fluorescently labelled PBAE. Once coated, the devices were either injected, in the case of MNs, or implanted as described in the explants. Proof of film release was confirmed by fluorescence microscopy (Nikon Eclipse TE200-U).

### 2.10. Transfection Efficiency Studies In Vitro

Transfection efficiency of the OM- and MM-PBAEs libraries was examined using human models of the main cell phenotypes dominating antigen presentation in the skin. Cells were seeded in a 96-well plate for 24 h prior to the transfection assay to reach an 80% confluence by the following day. 15,000 cells per well were seeded for all cell lines with the exemption of MoLCs (20,000 cells/well). Cells were incubated with 0.3 µg of pDNA per well encapsulated in the OM- and MM-PBAEs prepared at a 50:1 polymer-to-DNA ratio and triplicates were assayed for each formulation. Briefly, cells were washed with PBS and 200 µL of supplemented media including the polyplexes were added. Cells remained at 37 °C in 5% CO_2_ atmosphere until analysis. Untreated cells were used as negative control while the arginine-terminated PBAE CR3-C32 served as a positive control group. Lipofectamine 3000 (Thermo Fisher Scientific, Madrid, Spain) was also used as a commercial control and incubated at 0.1 µg µL^−1^ (concentration recommended by the manufacturer’s guidelines). 48 h post-transfection, gene delivery was analyzed by flow cytometry (BD LSR Fortessa™) and fluorescence microscopy (Nikon Eclipse TE2000-U). Transfection efficiency of PBAE-coated transdermal devices was examined in a similar fashion using 48-well plates where substrates were deposited gently on the wells.

### 2.11. Statistical Analysis

Statistical analysis was carried out with Graph-Pad Prism 8 (GraphPad Software). All error bars reported are SD unless otherwise indicated. Pairwise comparisons were performed using one-way Student’s *t*-tests. Differences between groups were considered significant at *p* values below 0.05 (ns = non-significant, * *p* < 0.05, ** *p* < 0.01, *** *p* < 0.001, **** *p* < 0.0001).

## 3. Results and Discussion

### 3.1. Decoration of Oligopeptide-Modified PBAEs with Mannose Moieties for APC-Targeting

**Figure 1:**
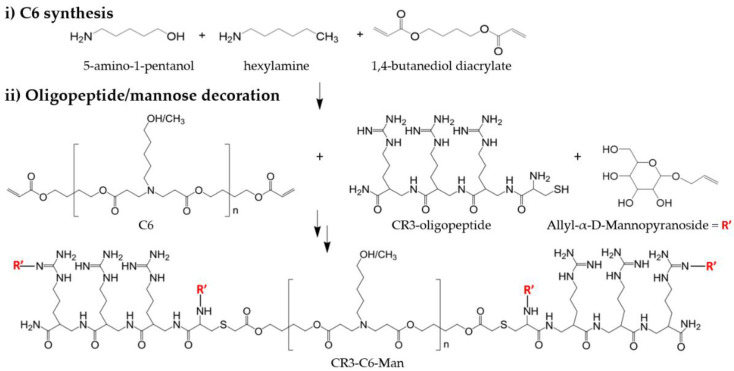
Synthesis of mannose-modified PBAEs derived from the C6 polymer. Decoration of the tri-arginine-modified PBAE is shown as an example. (**i**) Synthesis of the acrylate-terminated C6 PBAE via Michael addition. (**ii**) End-capping of the C6 polymer with oligopeptide moieties and mannosylation of oligopeptide-terminated PBAEs. R’ corresponds to predicted mannose targeting.

Our group has long confirmed end-capping of PBAEs with oligopeptide moieties as a powerful tool for specific cell targeting as decorated PBAEs display a viral-like tropism without necessitating ligand-mediated mechanisms [32,34,35,36,37]. Thus, we aimed to harness the OM-PBAE library derived from the top performing C6 polymer [32] as the starting point in the design of an APC-targeting vaccination technology. Given the scarcity of our cellular targets, especially LCs that only account for 1.86% of all epidermal cells [38], the former family of OM-C6 PBAEs was further iterated to generate polyplexes containing a mannose-targeting ligand to enhance the receptor-mediated delivery route via langerin and mannose receptor internalization. Polymerization of new mannosylated candidates was performed as described in Section 2. First, the C6 acrylate-terminated polymer was obtained by conjugate addition of hydrophilic/hydrophobic chains (5-amino-1-pentanol and hexylamine) in a stoichiometric proportion along with a slight excess of 1,4-butanediol (Figure 1i). Here, hydrophobization of the polymer backbone via addition of pendant hexyl groups is crucial to increase the stability and efficacy of resulting polyplexes [32,39,40,41], which are especially susceptible to premature disassembling due to unspecific interactions with glycosaminoglycans and other densely charged proteins present in the extracellular matrix of the skin. Then, the end-acrylate groups of the C6 polymer were further modified with oligopeptide moieties (CR3, CK3, CH3, or CD3 oligopeptides) using a molar excess to ensure full capping of the acrylate termini and avoid cytotoxicity issues (Figure 1ii). ^1^H-NMR spectroscopy confirmed the disappearance of acrylate signals after end-capping while peaks associated to oligopeptides surfaced in agreement with previously published data [16,17] (Figure S1, Supplementary Materials). Finally, both CR3- and CK3-terminated PBAEs were further reacted with allyl-α-D-Mannopyranoside (Figure 1ii) and where mannosylation was predicted to be determined by the delicate equilibrium between nucleophilicity and steric hindrance. Mannose decoration was only conducted on CR3- and CK3- PBAEs as they were the main constituents of future polyplexes.

### 3.2. Characterization of OM- and MM-PBAEs

In this work, plasmid DNA was chosen as a nucleic acid model to evaluate the transfection potential of the novel PBAE library. DNA-binding capability of OM-C6-PBAEs along with their mannosylated counterparts was evaluated by gel retardation assay. For clarity, polyplexes assessed in this and further assays were detailed in Table 1. Briefly, screened polyplexes were formulated with either a single polymer or a pair since our previous studies suggested that the properties of OM-PBAE are additive [16]. Overall, OM-PBAEs rendered polyplexes with higher complexation efficiencies if compared with those obtained from its predecessor, the C32 polymer [16], as DNA migration was impeded at ratios as low as 5:1 (*w*/*w*), suggesting that the inclusion of hydrophobic chains enhances complexation (Figure S2, Supplementary Materials). Results showed that mannosylation of OM-PBAEs did not interfere with DNA complexation as particles condensed at the same ratios as their non-mannosylated counterparts. Specifically, the presence of guanidinium groups and primary amines is known to facilitate the protonation of the CR3- and CK3-C6 polymers and so, favor the interaction with the negatively charged phosphate groups present in the nucleic acids. Contrarily, the limited protonation capacity of CH3-C6 polymer and the negatively charged aspartic acid residues in the CD3-C6 polymer was expected to hamper DNA complexation. Our results showed that most polyplexes formulated with single-end or mixtures of OM-and MM-PBAEs blocked DNA migration at the lowest ratio evaluated (5:1). Addition of anionic groups slightly delayed DNA complexation in K/H-Man and R/H-Man PBAEs.

**Figure 2:**
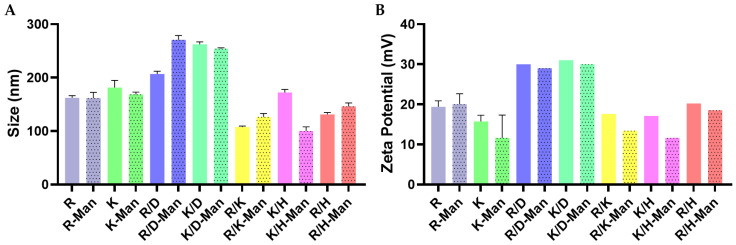
Analysis by Dynamic Light Scattering (DLS) of the average hydrodynamic diameter (**A**) and zeta-potential (**B**) distributions of polyplexes derived from the complexation of pDNA with OM-PBAEs and MM-PBAEs. Polydispersity index (PDI) values were lower than 0.3 for all measurements. Data are shown as mean and SD of triplicates (mean ± SD).

Complexes were further examined by DLS to evaluate their size and zeta potential. Screening of polyplexes prepared at a 50:1 polymer-to-DNA ratio using single-ended or mixtures of OM- and MM-PBAEs revealed that end-modifications had a notorious influence on their final physicochemical properties in agreement with our previously published data [16,17,32]. The hydrodynamic diameter of polyplexes derived from OM- and MM-PBAEs ranged from 100 to 250 nm and where mannosylation did not appear to impact their final size (Figure 2A). A subtle increase was observed for polyplexes formulated with the CD3-PBAE and which was hypothesized to originate from repulsive forces with the DNA (also negatively charged); thus, the increment of the particle size. 

Regarding their surface charge, results showed that decorative oligopeptides had a clear influence, where arginine-bearing polyplexes presented the most positive potential, followed by the pairs including lysine groups (Figure 2B). As expected, a sharp reduction of the surface charge was also confirmed when anionic moieties such as the aspartic acid were added, with R/D-derived polyplexes experiencing more than a 50% decrease in their charge if compared to R-derived polyplexes. Though not significant, a trend was observed as most of the polyplexes derived from MM-PBAEs presented lower surface charges if compared with their non-modified counterparts. Hence, our data hinted that the inclusion of mannose might be playing a shielding effect as suggested by others before [42], a desirable feature in further studies to limit the inherent cytotoxicity of densely charged particles [43].

### 3.3. Gene Delivery Studies in Professional APCs (Langerhans Cells)

Transfection efficiency of the newly synthesized families of OM- and MM-PBAEs was first tested in in vitro models of human Langerhans cells (hLCs) as key governors of antigen presentation in the epidermis layer [44]. Transfection studies were conducted in MUTZ-3 cells, a human myeloid-derived cell line that can be differentiated into a LC-like phenotype under cytokine induction, displaying a high resemblance with their primary counterparts in terms of phenotypic plasticity and functional/transcriptional profiles [45]. Following differentiation, MUTZ-3 cells express Langerin [46], which possesses mannose-binding affinity [20,47] and is hypothesized to favor receptor-mediated pathways of nanoparticle internalization. The use of an immortalized cell line was proposed due to the reported disadvantages in the development of standardized DC-vaccine technologies when monocyte-derived models are utilized, such as inter-/intra-donor variability and their limited availability [48].

**Figure 3:**
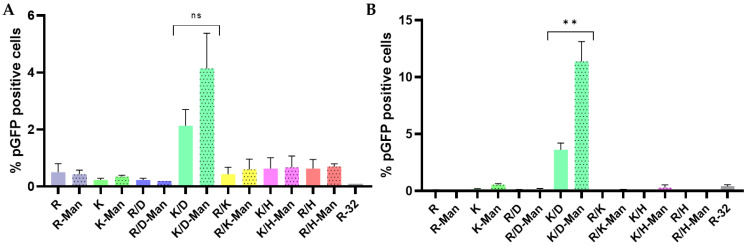
Assessment of PGFP-gene expression by flow cytometry in Langerhans-like cells. MUTZ-3 cells (**A**) and human MoLCs (**B**) after transfection using the library of OM- and MM-PBAEs. Cells were transfected by adding 0.3 µg pDNA/well at a 50:1 polymer:DNA weight ratio. Results are presented as mean and standard deviation of triplicates (mean ± SD). Statistical significance was compared between MM-PBAEs and their non-modified counterpart (ns = non-significant, ** *p* < 0.01).

Screening of the non-targeted and mannosylated-libraries of PBAEs revealed that only one formulation bearing both cationic and anionic oligopeptides was capable of inducing significant levels of gene expression in our hLCs model (Figure 3A). Here, polyplexes derived from lysine- and aspartic acid-modified PBAEs, along with the mannose-modified pair, achieved percentages of GFP-positive cells around 2 and 4%, respectively, while the rest of formulations barely mediated gene transfer. Our data confirmed the potential of oligopeptide-capping for cell-specific gene delivery while the addition of targeting ligands prompted even higher transfection efficiencies. We hypothesized that mannose was boosting the engulfment of polyplexes via clathrin- and scavenger receptor-dependent pathways as they are reported to dominate particle internalization in DCs when particles are sized around 250 nm or less [49], although mannose-decorated antigens can also be taken up through receptor-independent mechanisms such as macropinocytosis regardless of their size [50,51]. Concerning surface chemistry, both charge and the ligand organization pattern are known to have an impact on nanoparticle internalization by DCs [52]. In accordance with our previous data, we observed that charge distribution rather than the absolute zeta potential may be driving particle uptake [17], which could hint at why just a single formulation could effectively transfect LCs. We suspect that polyplexes decorated with lysine and aspartic acid residues might be displaying more positive groups in their surface that facilitated interaction with the negatively charged membrane of the cells. Also, the inclusion of anionic groups in a defined distribution is known to enhance the penetration and further endosomal scape [53]. This could explain why other polyplexes similarly formulated with cationic and anionic moieties (R/D and R/D-Man) barely mediated gene transference if compared with K/D- or K/D-Man-PBAEs. Indeed, a great diversity in the intracellular routing of lectin receptors such as Langerin has been reported, even within the same receptor depending on the ligand that it engages with [54]. All the above evidence shows the intricate mechanisms pursued by DCs to internalize nanoparticles as a function of their size, charge, and ligand motifs, which highlighted the need for further systematic screening to understand how nanoparticle internalization and endosomal scape influences in their final transfection efficiency.

Gene delivery efficiency of OM- and MM-PBAEs was also screened in human monocyte-derived Langerhans cells (MoLCs) obtained from healthy donors (Figure 3B). Despite the disadvantages mentioned herein, some support the use of monocyte-derived models as they can provide a better correlation with in vivo settings rather than immortalized APCs models, as the latter have been suggested to lose their avidness for nanoparticles [55]. Assessment of gene transfer revealed similar results when comparing both models of LCs. Once again, polyplexes formulated with the mixture of lysine- and acid aspartic-modified PBAEs (K/D and K/D-Man) outperformed the rest of their counterparts. Here, mannosylation had a bigger influence if compared to MUTZ3 cells, achieving a three-fold improved transfection efficiency. Overall, the percentages of transfected cells increased in MoLCs, highlighting the need to account for model variability in gene delivery studies.

### 3.4. Gene Delivery Studies in Non-Professional APCs

**Figure 4:**
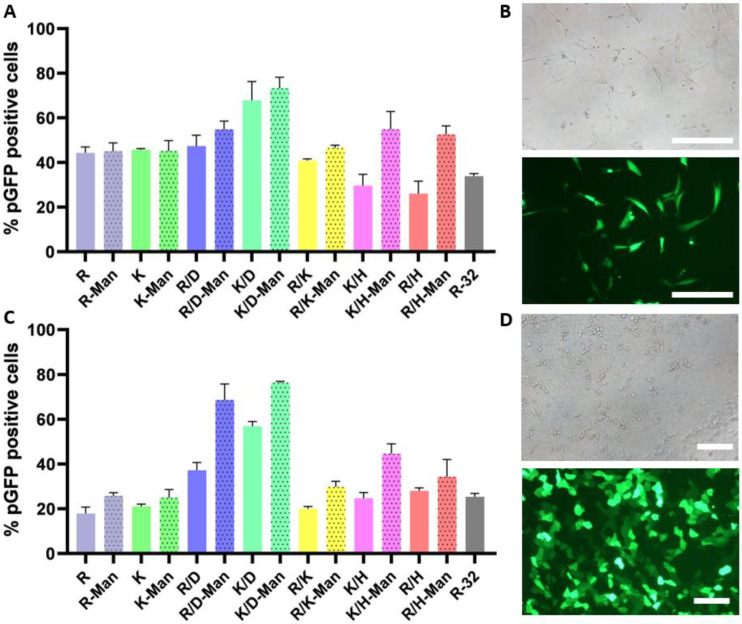
Evaluation of PGFP gene expression in human immortalized fibroblasts (hNDFs) by flow cytometry (**A**) and microscopy (**B**). Scale bar = 100 µm. Quantification of GFP-positive cells following transfection of HaCaT cells with OM- and MM-derived polyplexes by flow cytometry (**C**). Light and fluorescence microscopy of immortalized keratinocytes (**D**). Scale bar = 50 µm. All cell lines were transfected with the OM- and MM-PBAE libraries by adding 0.3 µg pDNA/well at a 50:1 polymer-to-pDNA weight ratio. Arginine-terminated 32 PBAE was used as positive control. Images depict cells transfected with K/D-Man-derived polyplexes. Results are presented as mean and standard deviation of triplicates (mean ± SD).

Dendritic cells including LCs are known to dominate antigen presentation in the skin epidermis and dermis milieu [56], yet other accessory cells such as fibroblasts or keratinocytes are also orchestrators in the elicitation of immune responses via secretion of co-stimulatory molecules or by acting as “non-professional” antigen-presenting cells, as they are capable of priming naïve-reactive T cells [57,58,59]. Therefore, we further investigated the transfection capacity of the OM- and MM-PBAEs loaded with pDNA when incubated with human fibroblast and keratinocyte models in vitro. The inclusion of mannose moieties to the PBAEs backbone was proven to be an effective synthetic strategy to boost transfection levels in human normal fibroblast cells (hNDF) (Figure 4A,B). Results suggested that mannosylation prompted superior gene delivery in most formulations, with the greatest improvement observed in histidine-bearing polyplexes, which went from registering the worst performance to doubling their transfection levels after mannosylation. We hypothesized that the expression of polysaccharide-binding receptors on the surface of fibroblasts may be favoring cellular uptake as they can internalize glycosylated ligands from the extracellular space [60,61] and in turn, favor specific routes for polyplexes to enter into the intracellular space. In this model, oligopeptide-decoration had a timid impact if comparing the performance of the formulations except for K/D-PBAEs and its mannosylated counterpart, which generated the highest levels of gene expression.

Transfection assays using HaCaT cells revealed a similar trend as when using the fibroblast model with polyplexes derived from MM-PBAEs inducing overall greater levels of gene expression (Figure 4C,D). Again, polyplexes formulated with mixtures of OM- and MM-PBAEs –especially when containing both cationic and anionic oligopeptides– outperformed those that were single-ended, reaching a 75% percentage of GFP-positive cells for the K/D-Man PBAEs. Our results confirmed the additive influence of both mannosylation and oligopeptide-modification which dramatically enhanced the performance of polyplexes, rising from 20% of transfected cell to more than 70% for top formulations. Expression of mannose-binding receptors such as the mannose receptor (MR, CD206) by keratinocytes may tentatively support the key role of mannosylation in prompting superior gene transfer [60], yet further inhibition and nanoparticle internalization studies would be required to elucidate the contribution of each element to their final performance.

### 3.5. Surface-Mediated Gene Delivery Using Multilayer Polyelectrolyte PBAE Films

**Figure 5:**
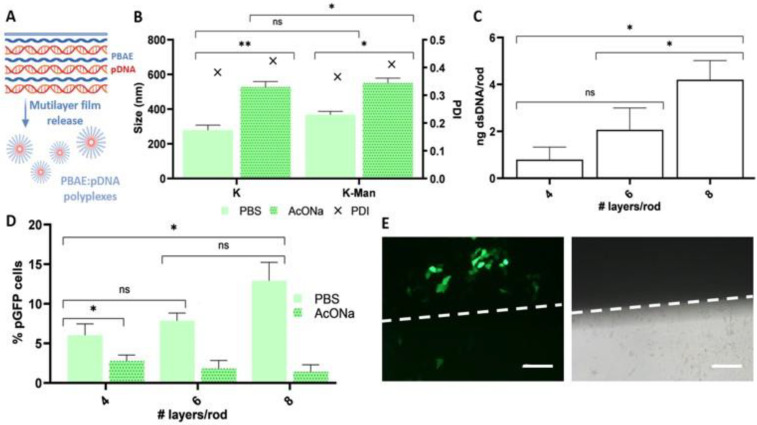
Schematic representation of in situ polyplex formation from PBAE:pDNA polyelectrolyte films (**A**). Hydrodynamic diameter (left y axis; bars) and PDI (right y axis; symbols) of polyplexes derived from K and K-Man-PBAEs (2:1 polymer-to-DNA ratio) upon release from PCL substrates. Polyelectrolyte films were deposited using PBS or AcONa as dilution buffer (**B**). Fluorescence quantification of dsDNA from eroded films prepared with PBS and quantified with the High Sensitivity Quant-iT™ dsDNA Assay Kit (**C**). Evaluation of pGFP expression in HaCaT cells following transfection with PCL rods coated with K-Man-PBAE:pDNA films by flow cytometry (**D**). Fluorescence versus bright field microscopy served to confirm local transfection. Dashed lines delimit area in contact with films. Scale bar = 50 µm (**E**). Data are represented as mean ± SD (ns = non-significant, * *p* < 0.05, ** *p* < 0.01).

We next studied the potential of OM- and MM-PBAEs to transfer genes when deposited in the form of hydrolytically degradable films as these can generate polyplexes in situ upon release (Figure 5A). Characterization of the PBAE:pDNA polyelectrolyte films was first conducted on polymeric rods with a surface area of ≈100 mm^2^ that resembled an existing subcutaneous device, contraceptive implants, as their easy fabrication offered a good opportunity to investigate the performance of the polymers in a translational device. First, in situ formation of polyplexes following film erosion from the rods was studied by DLS. In consonance with others [24,33,62], we reported a high polydispersity in all the samples (PDI > 0.3) and where aggregates ranging from 250 to 550 nm were observed regardless of the buffering solution utilized during their fabrication (Figure 5B). Mannosylation did not appear to influence the final size of the aggregates, whereas the dipping buffer did, since significantly higher aggregates were engendered when using AcONa. Next, we quantified pDNA deposition on the polymeric rods when coated with an increasing number of bilayers. Fluorescence analysis of supernatants recovered from eroded PBAE:pDNA films showed a correlation between the mass of pDNA deposited and the number of bilayers, hinting that film growth was occurring, although statistically significant differences could not be claimed among all groups (Figure 5C). 

Finally, we evaluated transfection efficiency of the polyelectrolyte films as a function of bilayer numbers using a permissive cell line. Flow cytometry results following transfection assays first pointed at the buffer as a critical parameter in the mediation of gene delivery. Our data showed that the percentage of transfected cells when using AcONa-derived films was significantly lower if compared to those achieved when fabricating the films with PBS (Figure 5D). A trend was also evidenced between the number of bilayers and the percentage of transfected cells, where the highest number of GFP-positive cells was registered for those rods accumulating more layers. As predicted, cells located under the functionalized substrates were preferentially transfected if compared to those in distant regions of the well (Figure 5E).

### 3.6. PBAE-Based Delivery Systems Can Be Integrated with Transdermal Devices for Dermal Delivery

**Figure 6:**
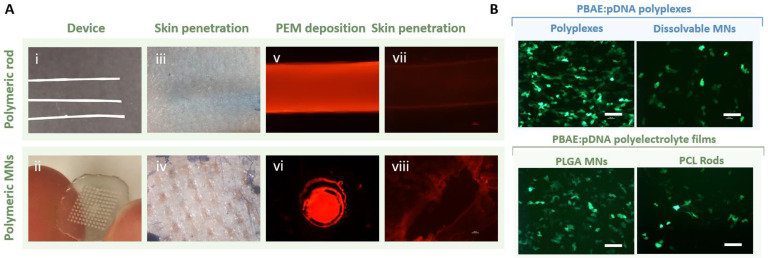
Ex vivo characterization of medical devices for prospective vaccine delivery using PBAE polyelectrolyte films. Capacity of transdermal devices to effectively penetrate the skin was tested using cadaveric skin explants from C57BL/6 healthy mice along with film deposition from rods (**top panel**) or solid PLGA-based MNs (**bottom panel**) (**A**). Fluorescence microscopy following transfection with the K/D and K/D-Man polymers as PBAE:pDNA polyplexes (**top**) and PBAE:pDNA films (**bottom**) in HaCaT cells (**B**). Scale bar = 50 µm.

Besides from polymeric rods, we broadly explored the use of needle-free technologies such as microneedles for PBAE-based gene delivery in contrast to invasive approaches (Figure 6A(i,ii)). The non-invasive and pain-free nature of MNs is known to facilitate high patient compliance and minimize the risk of infections while also enhancing the efficacy and tolerability of the therapeutic by exposing it to the intended molecular targets; [63] here, APCs. We first examined their capacity to disrupt the most outer layers of the skin using cadaveric mice explants as an ex vivo model. We confirmed that both polymeric rods and MN patches could be easily administrated (Figure 6A), yet we predicted that the unavoidable invasiveness of the first could be a limiting factor for widespread adoption and patient acceptance. Contrarily, MN administration was a user-friendly procedure that managed to effectively pierce the skin as evidenced by the presence of micro conduits in the epidermis (Figure 6A(iv)). We next examined whether PBAE:pDNA polyelectrolyte films would be released in the skin upon administration. Here, both polymeric rods and MNs were coated as described with bilayers of fluorescently-labelled polymer and pDNA and film deposition was confirmed by fluorescent microscopy (Figure 6A(v,vi)). Next, the devices were implanted in the skin explants and imaged. Film release was especially evident for those explants pierced with the MNs, since the polymer could be observed all over the margins of the micro conduit (Figure 6A(viii)), confirming the adequacy of these approaches for future in vivo application.

Given the potential of OM- and MM-PBAE candidates to induce both nanoparticle- and surface-mediated transfection, we next compared their performance in vitro when integrated with various devices used for local vaccination. Specifically, we investigated PBAE transfection efficacy when delivery as polyplexes either on their own or embedded in dissolvable MNs or, alternatively, when deposited onto solid MNs and polymeric rods as polyelectrolyte films. Results for polyplex-mediated delivery confirmed that polyplexes embedded within the matrix of dissolvable poly(vinyl alcohol) MNs could efficiently transfect HaCaT cells, although the total number of positive events was significantly reduced if compared to that when using PBAE polyplexes as the control group (Figure 6B, top). PBAE:pDNA polyelectrolyte films were also confirmed to induce gene expression when deposited into surfaces other than the rods. Superior transfection levels were reported for coated polymeric MNs if compared to those promoted by coated PCL rods, which was attributed to the increased surface area of the MNs provided by the patch indentations and the expanded area that was in direct contact with the cells (Figure 6B, bottom).

### 3.7. Targeted LCs Transfection with OM-/MM-PBAEs Induce Superior Gene Delivery for DNA Vaccination

**Figure 7:**
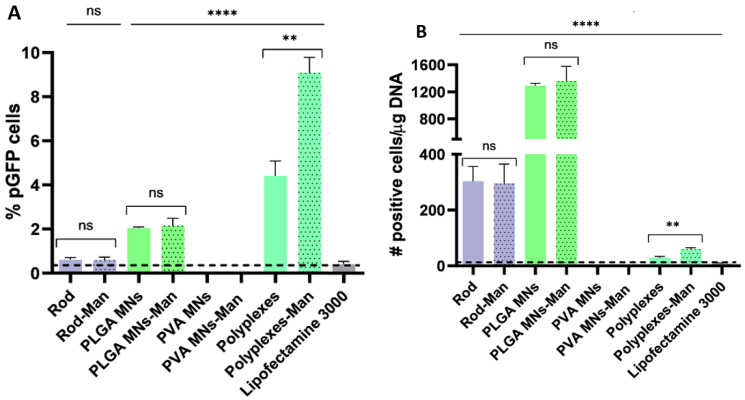
Screening of pGFP expression in MoLCs cells by flow cytometry following transfection with PBAE-based delivery systems (**A**). Number of transfected cells theoretically normalized to the amount of pDNA added per well during transfection (**B**). Values from our previous dsDNA quantification assay in rods were extrapolated for coated MNs. Data are represented as mean ± SD. Unless stated, statical significance was determined against Lipofectamine3000 (dashed line) as the control group (ns = non-significant, ** *p* < 0.01, **** *p* < 0.0001).

Lastly, we confirmed the transfection efficiency of the PBAE-based delivery systems using human-derived Langerhans cells (MoLCs), our main cellular target. Evaluation of GFP expression by flow cytometry confirmed the superiority of polyplexes (Figure 7A), where the K/D-Man-PBAEs surpassed all the approaches screened and registered a ≈10-fold increased efficiency if compared to an advanced commercial vector (Lipofectamine 3000). Contrastingly, gene expression was not observed when using dissolvable MNs loaded with PBAEs, despite adding an equal amount of polyplexes as for the control group. PBAE:pDNA films also triggered notable levels of gene transference when deposited in both polymeric rods and MNs, which rivalled or even outperformed those obtained by the commercial control. No differences in gene expression were observed when using OM-PBAEs films (K/D) or their mannosylated counterparts (K/D-Man), evidencing the need for further studies to fully address the role of targeting moieties in surface-mediated transfection. Overall, levels of gene expression induced by PBAE:pDNA films were significantly lower if compared to those mediated by the corresponding PBAE-derived polyplexes. Reduced transfection percentages were attributed to the limited deposition yield of pDNA being 100-times lower if normalized to the µg of pDNA added per well and compared to the experimental amounts added per well when using polyplexes. Nevertheless, we predicted that the low deposition yield could be addressed in the future as it has been reported that up to 40 bilayers can be piled [25]. Also, the reduced surface area of the substrates limited the number of cells in close contact with them for effective transfection, as their area just accounted for a tenth of the total cell growth (for polymeric rods) or half (for MNs). Using the same data as for Figure 7A, we also represented the number of transfected cells as a function of µg of DNA per well instead of total positive cells to obtain a more truthful insight into the actual transfection yields (Figure 7B). Theoretically, delivery using PBAE:pDNA films appeared to dominate over nanoparticle-based strategies if normalizing transfection yields to mass of pDNA added per well, and where coated MNs induced the highest levels of gene delivery with a 100-fold increased efficiency. After them, polymeric rods also promoted notable levels of transfection, followed by polyplexes in the last place. More importantly, all PBAE-derived systems would appear to surpass the commercial standard for transfection (Lipofectamine 3000). While strictly theoretical, these results support the potential of polyelectrolyte films for solvent-free gene vaccination, especially when integrated with non-invasive devices such as MNs. We, and others, have confirmed the therapeutic merit of PBAE-derived systems for transdermal drug delivery in the context of gene vaccination [24,25] but also for the delivery of other nucleic acid immunomodulators such as TLR agonist [30]. When integrated with microneedles, either as PEMS or as polyplexes, PBAE-derived systems induced potent responses for immunization and immunomodulation. In future studies, we will rationally select PBAE-derived vectors to favor certain mechanisms for antigen presentation and in turn, the concomitant induction of helper immune responses with antibody production and cytotoxic cellular responses for the generation of long-term and robust immunity.

## 4. Conclusions

Superior vectors targeting the transdermal reservoir of APCs are still needed to prompt long-lasting and robust immune responses following nucleic acid vaccination. In this report, the authors engineered a new library of PBAEs terminated with oligopeptides and a mannose ligand for cell-specific targeting of LCs residing in the dermal compartment. Screening of cell transfection efficiency using human APCs attested the superiority of polyplexes bearing both lysine- and aspartic acid-terminated PBAEs. Chemical linkage of oligopeptide moieties appeared to be the driving force governing cell-specific targeting while mannosylation proved to augment the PBAEs transfection yield in both professional and accessory APCs. Deposition of the newly synthesized OM- and MM-PBAEs as polyelectrolyte films promoted superior levels of surface gene transfection, opening up potential avenues for non-invasive vaccination using devices such as MNs to simplify waste disposal while promising solvent-free and cold chain independency. Our thorough in vitro examination set the bases for prospective in vivo vaccination studies, where the use of an outperforming delivery vector could surmount the current caveats associated with transcutaneous immunization.

## Figures and Tables

**Table 1:** List of screened polyplexes derived from single or multiple OM- and MM-PBAEs (Man = mannosylated).

Single OM-/MM-Polyplexes			
Nomenclature	Oligopeptide 1		
R	Cys + 3Arg		
K	Cys + 3Lys		
R-Man	Cys + 3Arg-man		
K-Man	Cys + 3Lys-man		
**Multiple OM-/MM-polyplexes**			
**Nomenclature**	**Oligopeptide 1**	**Oligopeptide 2**	**Ratio**
R/D	Cys + 3Arg	Cys + 3Asp	70:30
K/D	Cys + 3Lys	Cys + 3Asp	70:30
R/K	Cys + 3Arg	Cys + 3Lys	50:50
K/H	Cys + 3Lys	Cys + 3His	50:50
R/H	Cys + 3Arg	Cys + 3His	50:50
R/D-Man	Cys + 3Arg-man	Cys + 3Asp	70:30
K/D-Man	Cys + 3Lys-man	Cys + 3Asp	70:30
R/K-Man	Cys + 3Arg-man	Cys + 3Lys-man	50:50
R/H-Man	Cys + 3Arg-man	Cys + 3His	50:50
K/H-Man	Cys + 3Lys-man	Cys + 3His	50:50

## Data Availability

Data presented in this study are available upon request.

## Supplementary Materials

The following supporting information can be downloaded at: https://www.mdpi.com/article/10.3390/pharmaceutics15041262/s1, Figure S1: NMR spectra; Figure S2: Gel retardation assays with OM- and MM-PBAEs.

## References

[1] Kulkarni J.A., Witzigmann D., Thomson S.B., Chen S., Leavitt B.R., Cullis P.R., van der Meel R. (2021). The Current Landscape of Nucleic Acid Therapeutics. Nat. Nanotechnol..

[2] Barbier A.J., Jiang A.Y., Zhang P., Wooster R., Anderson D.G. (2022). The Clinical Progress of MRNA Vaccines and Immunotherapies. Nat. Biotechnol..

[3] Chaudhary N., Weissman D., Whitehead K.A. (2021). MRNA Vaccines for Infectious Diseases: Principles, Delivery and Clinical Translation. Nat. Rev. Drug. Discov..

[4] Brisse M., Vrba S.M., Kirk N., Liang Y., Ly H. (2020). Emerging Concepts and Technologies in Vaccine Development. Front. Immunol..

[5] Rosa S.S., Prazeres D.M.F., Azevedo A.M., Marques M.P.C. (2021). MRNA Vaccines Manufacturing: Challenges and Bottlenecks. Vaccine.

[6] Pardi N., Hogan M.J., Porter F.W., Weissman D. (2018). MRNA Vaccines—A New Era in Vaccinology. Nature.

[7] Hettinga J., Carlisle R. (2020). Vaccination into the Dermal Compartment: Techniques, Challenges, and Prospects. Vaccines.

[8] Karande P., Mitragotri S. (2010). Transcutaneous Immunization: An Overview of Advantages, Disease Targets, Vaccines, and Delivery Technologies. Annu. Rev. Chem. Biomol. Eng..

[9] Prausnitz M.R., Langer R. (2008). Transdermal Drug Delivery. Nat. Biotechnol..

[10] Valdivia-Olivares R.Y., Rodriguez-Fernandez M., Álvarez-Figueroa M.J., Kalergis A.M., González-Aramundiz J.V. (2021). The Importance of Nanocarrier Design and Composition for an Efficient Nanoparticle-Mediated Transdermal Vaccination. Vaccines.

[11] Pielenhofer J., Sohl J., Windbergs M., Langguth P., Radsak M.P. (2020). Current Progress in Particle-Based Systems for Transdermal Vaccine Delivery. Front. Immunol..

[12] Anderson D.G., Lynn D.M., Langer R. (2003). Semi-Automated Synthesis and Screening of a Large Library of Degradable Cationic Polymers for Gene Delivery. Angew. Chem. Int. Ed..

[13] Green J.J., Chiu E., Leshchiner E.S., Shi J., Langer R., Anderson D.G. (2007). Electrostatic Ligand Coatings of Nanoparticles Enable Ligand-Specific\rGene Delivery to Human Primary Cells. Nano Lett..

[14] Zugates G.T., Tedford N.C., Zumbuehl A., Jhunjhunwala S., Kang C.S., Griffith L.G., Lauffenburger D.A., Langer R., Anderson D.G. (2007). Gene Delivery Properties of End-Modified Poly (Beta-Amino Ester)s. Bioconjug. Chem..

[15] Sunshine J., Green J.J., Mahon K.P., Yang F., Eltoukhy A.A., Nguyen D.N., Langer R., Anderson D.G. (2009). Small-Molecule End-Groups of Linear Polymer Determine Cell-Type Gene-Delivery Efficacy. Adv. Mater..

[16] Segovia N., Dosta P., Cascante A., Ramos V., Borrós S. (2014). Oligopeptide-Terminated Poly (b-Amino Ester) s for Highly Efficient Gene Delivery and Intracellular Localization. Acta Biomater..

[17] Dosta P., Segovia N., Cascante A., Ramos V., Borrós S. (2015). Surface Charge Tunability as a Powerful Strategy to Control Electrostatic Interaction for High Efficiency Silencing, Using Tailored Oligopeptide-Modified Poly(Beta-Amino Ester)s (PBAEs). Acta Biomater..

[18] Stoitzner P., Sparber F., Tripp C.H. (2010). Langerhans Cells as Targets for Immunotherapy against Skin Cancer. Immunol. Cell. Biol..

[19] Romani N., Flacher V., Tripp C.H., Sparber F., Ebner S., Stoitzner P. (2012). Targeting Skin Dendritic Cells to Improve Intradermal Vaccination. Curr. Top. Microbiol. Immunol..

[20] Valladeau J., Ravel O., Dezutter-Dambuyant C., Moore K., Kleijmeer M., Liu Y., Duvert-Frances V., Vincent C., Schmitt D., Davoust J. (2000). Langerin, a Novel C-Type Lectin Specific to Langerhans Cells, Is an Endocytic Receptor That Induces the Formation of Birbeck Granules. Immunity.

[21] Irache J.M., Salman H.H., Gamazo C., Espuelas S. (2008). Mannose-Targeted Systems for the Delivery of Therapeutics. Expert. Opin. Drug. Deliv..

[22] Iqbal S., Qu Y., Dong Z., Zhao J., Khan A.R., Rehman S., Zhao Z. (2020). Poly (Β-Amino Esters) Based Potential Drug Delivery and Targeting Polymer; An Overview and Perspectives. Eur. Polym. J..

[23] Liu Y., Li Y., Keskin D., Shi L. (2019). Poly (β-Amino Esters): Synthesis, Formulations, and Their Biomedical Applications. Adv. Healthc. Mater..

[24] Demuth P.C., Min Y., Huang B., Kramer J.A., Miller A.D., Barouch D.H., Hammond P.T., Irvine D.J. (2013). Polymer Multilayer Tattooing for Enhanced DNA Vaccination. Nat. Mater..

[25] Su X., Kim B.S., Kim S.R., Hammond P.T., Irvine D.J. (2009). Layer-by-Layer-Assembled Multilayer Films for Transcutaneous Drug and Vaccine Delivery. ACS Nano.

[26] Demuth P.C., Su X., Samuel R.E., Hammond P.T., Irvine D.J. (2010). Nano-Layered Microneedles for Transcutaneous Delivery of Polymer Nanoparticles and Plasmid DNA. Adv. Mater..

[27] Guillot A.J., Cordeiro A.S., Donnelly R.F., Montesinos M.C., Garrigues T.M., Melero A. (2020). Microneedle-based Delivery: An Overview of Current Applications and Trends. Pharmaceutics.

[28] Yang J., Liu X., Fu Y., Song Y. (2019). Recent Advances of Microneedles for Biomedical Applications: Drug Delivery and Beyond. Acta Pharm. Sin. B.

[29] Kim Y.-C., Park J.H., Prausnitz M.R. (2012). Microneedles for Drug and Vaccine Delivery. Adv. Drug. Deliv. Rev..

[30] Dosta P., Puigmal N., Cryer A.M., Rodríguez A.L., Scott E., Weissleder R., Miller M.A., Artzi N. (2023). Polymeric Microneedles Enable Simultaneous Delivery of Cancer Immunomodulatory Drugs and Detection of Skin Biomarkers. Theranostics.

[31] Du G., Hathout R.M., Nasr M., Nejadnik M.R., Tu J., Koning R.I., Koster A.J., Slütter B., Kros A., Jiskoot W. (2017). Intradermal Vaccination with Hollow Microneedles: A Comparative Study of Various Protein Antigen and Adjuvant Encapsulated Nanoparticles. J. Control. Release.

[32] Dosta P., Ramos V., Borrós S. (2018). Stable and Efficient Generation of Poly(β-Amino Ester)s for RNAi Delivery. Mol. Syst. Des. Eng..

[33] Bechler S.L., Lynn D.M. (2012). Characterization of Degradable Polyelectrolyte Multilayers Fabricated Using DNA and a Fluorescently-Labeled Poly(B-Amino Ester): Shedding Light on the Role of the Cationic Polymer in Promoting Surface-Mediated Gene Delivery. Biomacromolecules.

[34] Dosta P., Tamargo I., Ramos V., Kumar S., Kang D.W., Borrós S., Jo H. (2021). Delivery of Anti-MicroRNA-712 to Inflamed Endothelial Cells Using Poly(β-Amino Ester) Nanoparticles Conjugated with VCAM-1 Targeting Peptide. Adv. Healthc. Mater..

[35] Fornaguera C., Guerra-rebollo M., Lázaro M.Á., Castells-sala C., Meca-cortés O., Ramos-pérez V., Cascante A., Rubio N., Blanco J., Borrós S. (2018). MRNA Delivery System for Targeting Antigen-Presenting Cells In Vivo. Adv. Healthc. Mater..

[36] Fornaguera C., Guerra-Rebollo M., Lázaro M.Á., Cascante A., Rubio N., Blanco J., Borrós S. (2019). In Vivo Retargeting of Poly(Beta Aminoester) (OM-PBAE) Nanoparticles Is Influenced by Protein Corona. Adv. Healthc. Mater..

[37] Dosta P., Demos C., Ramos V., Kang D.W., Kumar S., Jo H., Borrós S. (2021). Delivery of SiRNA to Endothelial Cells In Vivo Using Lysine/Histidine Oligopeptide-Modified Poly(β-Amino Ester) Nanoparticles. Cardiovasc. Eng. Technol..

[38] Bauer J., Bahmer F.A., Wörl J., Neuhuber W., Schuler G., Fartasch M. (2001). A Strikingly Constant Ratio Exists Between Langerhans Cells and Other Epidermal Cells in Human Skin. A Stereologic Study Using the Optical Disector Method and the Confocal Laser Scanning Microscope. J. Investig. Dermatol..

[39] Sunshine J.C., Akanda M.I., Li D., Kozielski K.L., Green J.J. (2011). Effects of Base Polymer Hydrophobicity and End-Group Modification on Polymeric Gene Delivery. Biomacromolecules.

[40] Liu Z., Zhang Z., Zhou C., Jiao Y. (2010). Hydrophobic Modifications of Cationic Polymers for Gene Delivery. Prog. Polym. Sci..

[41] Incani V., Lavasanifar A., Uludağ H. (2010). Lipid and Hydrophobic Modification of Cationic Carriers on Route to Superior Gene Vectors. Soft Matter.

[42] Jones C.H., Chen M., Gollakota A., Ravikrishnan A., Zhang G., Lin S., Tan M., Cheng C., Lin H., Pfeifer B.A. (2015). Structure−Function Assessment of Mannosylated Poly(β-Amino Esters) upon Targeted Antigen Presenting Cell Gene Delivery. Biomacromolecules.

[43] Fröhlich E. (2012). The Role of Surface Charge in Cellular Uptake and Cytotoxicity of Medical Nanoparticles. Int. J. Nanomed..

[44] Kaplan D.H. (2010). In Vivo Function of Langerhans Cells and Dermal DC. Trends Immunol..

[45] Larsson K., Lindstedt M., Borrebaeck C.A.K. (2006). Functional and Transcriptional Profiling of MUTZ-3, a Myeloid Cell Line Acting as a Model for Dendritic Cells. Immunology.

[46] Masterson A.J., Sombroek C.C., de Gruijl T.D., Graus Y.M.F., van der Vliet H.J.J., Lougheed S.M., van den Eertwegh A.J.M., Pinedo H.M., Scheper R.J. (2002). MUTZ-3, a Human Cell Line Model for the Cytokine-Induced Differentiation of Dendritic Cells from CD34+precursors. Blood.

[47] Tada Y., Riedl E., Lowenthal M.S., Liotta L.A., Briner D.M., Crouch E.C., Udey M.C. (2006). Identification and Characterization of Endogenous Langerin Ligands in Murine Extracellular Matrix. J. Investig. Dermatol..

[48] Santegoets S.J.A.M., van den Eertwegh A.J.M., van de Loosdrecht A.A., Scheper R.J., de Gruijl T.D. (2008). Human Dendritic Cell Line Models for DC Differentiation and Clinical DC Vaccination Studies. J. Leukoc. Biol..

[49] Zhang L.W., Bäumer W., Monteiro-Riviere N.A. (2011). Cellular Uptake Mechanisms and Toxicity of Quantum Dots in Dendritic Cells. Nanomedicine.

[50] Hillaireau H., Couvreur P. (2009). Nanocarriers’ Entry into the Cell: Relevance to Drug Delivery. Cell. Mol. Life Sci..

[51] Manzanares D., Ceña V. (2020). Endocytosis: The Nanoparticle and Submicron Nanocompounds Gateway into the Cell. Pharmaceutics.

[52] Jia J., Zhang Y., Xin Y., Jiang C., Yan B., Zhai S. (2018). Interactions Between Nanoparticles and Dendritic Cells: From the Perspective of Cancer Immunotherapy. Front. Oncol..

[53] Verma A., Uzun O., Hu Y., Hu Y., Han H., Watson N., Chen S., Irvine D.J., Stellacci F. (2008). Surface-Structure-Regulated Cell-Membrane Penetration by Monolayer-Protected Nanoparticles. Nat. Mater..

[54] Keler T., Ramakrishna V., Fanger M.W. (2004). Mannose Receptor-Targeted Vaccines. Expert. Opin. Biol. Ther..

[55] Silva J.M., Vandermeulen G., Oliveira V.G., Pinto S.N., Rodrigues C., Salgado A., Afonso C.A., Viana A.S., Jérôme C., Silva L.C. (2014). Development of Functionalized Nanoparticles for Vaccine Delivery to Dendritic Cells: A Mechanistic Approach. Nanomedicine.

[56] Liard C., Munier S., Joulin-Giet A., Bonduelle O., Hadam S., Duffy D., Vogt A., Verrier B., Combadière B. (2012). Intradermal Immunization Triggers Epidermal Langerhans Cell Mobilization Required for CD8 T-Cell Immune Responses. J. Investig. Dermatol..

[57] Heath W.R., Carbone F.R. (2013). The Skin-Resident and Migratory Immune System in Steady State and Memory: Innate Lymphocytes, Dendritic Cells and T Cells. Nat. Immunol..

[58] Kündig T.M., Bachmann M.F., DiPaolo C., Simard J.J., Battegay M., Lother H., Gessner A., Kühlcke K., Ohashi P.S., Hengartner H. (1995). Fibroblasts as Efficient Antigen-Presenting Cells in Lymphoid Organs. Science.

[59] Kim B.S., Miyagawa F., Cho Y.-H., Bennett C.L., Clausen B.E., Katz S.I. (2009). Keratinocytes Function as Accessory Cells for Presentation of Endogenous Antigen Expressed in the Epidermis. J. Investig. Dermatol..

[60] Jones C.H., Chen M., Ravikrishnan A., Reddinger R., Zhang G., Hakansson A.P., Pfeifer B.A. (2014). Mannosylated Poly (Beta-Amino Esters) for Targeted Antigen Presenting Cell Immune Modulation. Biomaterials.

[61] Sheikh H., Yarwood H., Ashworth A., Isacke C.M. (2000). Endo180, an Endocytic Recycling Glycoprotein Related to the Macrophage Mannose Receptor Is Expressed on Fibroblasts, Endothelial Cells and Macrophages and Functions as a Lectin Receptor. J. Cell. Sci..

[62] Jewell C.M., Zhang J., Fredin N.J., Lynn D.M. (2005). Multilayered Polyelectrolyte Films Promote the Direct and Localized Delivery of DNA to Cells. J. Control. Release.

[63] Lee K.J., Jeong S.S., Roh D.H., Kim D.Y., Choi H.K., Lee E.H. (2020). A Practical Guide to the Development of Microneedle Systems—In Clinical Trials or on the Market. Int. J. Pharm..

