# Supplementary material for: Poly(β-amino ester)s-Based Delivery Systems for Targeted Transdermal Vaccination
Source: Pharmaceutics. 2023 Apr 17;15(4):1262. doi: 10.3390/pharmaceutics15041262 (PMC10143071; doi:10.3390/pharmaceutics15041262)
Supplement: Supplementary file 1 [file pharmaceutics-15-01262-s001.zip › pharmaceutics-2327201-supplementary.pdf]

## Figure S1

NMR Spectra:

CR3-Man-C6

**$^{1}$H-NMR:** (400 MHz, CD3OD, TMS) (ppm): δ = 4.41-4.33 (br, NH2-C(=O)-CH-NH-C(=O)-CH-NH-C(=O)-CH- NH-C(=O)-CH-CH2-, 4.11 (t, CH2-CH2-O), 3.55 (t, CH2-CH2-OH), 3.22 (br, NH2-C(=NH)-NH-CH2-, OH- (CH2)4-CH2-N-), 3.04 (t, CH2-CH2-N-), 2.82 (dd, -CH2-S-CH2), 2.48 (br, -N-CH2-CH2-C(=O)-O), 1.90 (m, NH2-C(=NH)-NH-(CH2)2-CH2-CH-), 1.73 (br, -O-CH2-CH2-CH2-CH2-O), 1.69 (m, NH2-C(=NH)-NH-CH2- CH2-CH2-), 1.56 (br, -CH2-CH2-CH2-CH2-OH), 1.39 (br, -N-(CH2)2-CH2-(CH2)2-OH), 0.88 (t, CH2-CH2- CH3).

CK3-Man-C6

**$^{1}$H-NMR:** (400 MHz, CD3OD, TMS) (ppm): δ = 4.38-4.29 (br, NH2-(CH2) 4-CH-), 4.13 (t, CH2-CH2-O-),3.73 (br,NH2-CH-CH2-S-), 3.55 (t, CH2-CH2-OH), 2.94 (br, CH2-CH2-N-, NH2-CH2-(CH2)3-CH-), 2.81 (dd, -CH2- S-CH2), 2.57 (br, -N-CH2-CH2-C(=O)-O), 1.85 (m, NH2-(CH2)3-CH2-CH-), 1.74 (br, -O-CH2-CH2-CH2-CH2- O), 1.68 (m, NH2-CH2-CH2-(CH2) 2-CH-), 1.54 (br, -CH2-CH2-CH2-CH2-OH), 1.37 (br, -N-(CH2)2-CH2- (CH2)2-OH), 0.88 (t, CH2-CH2-CH3).

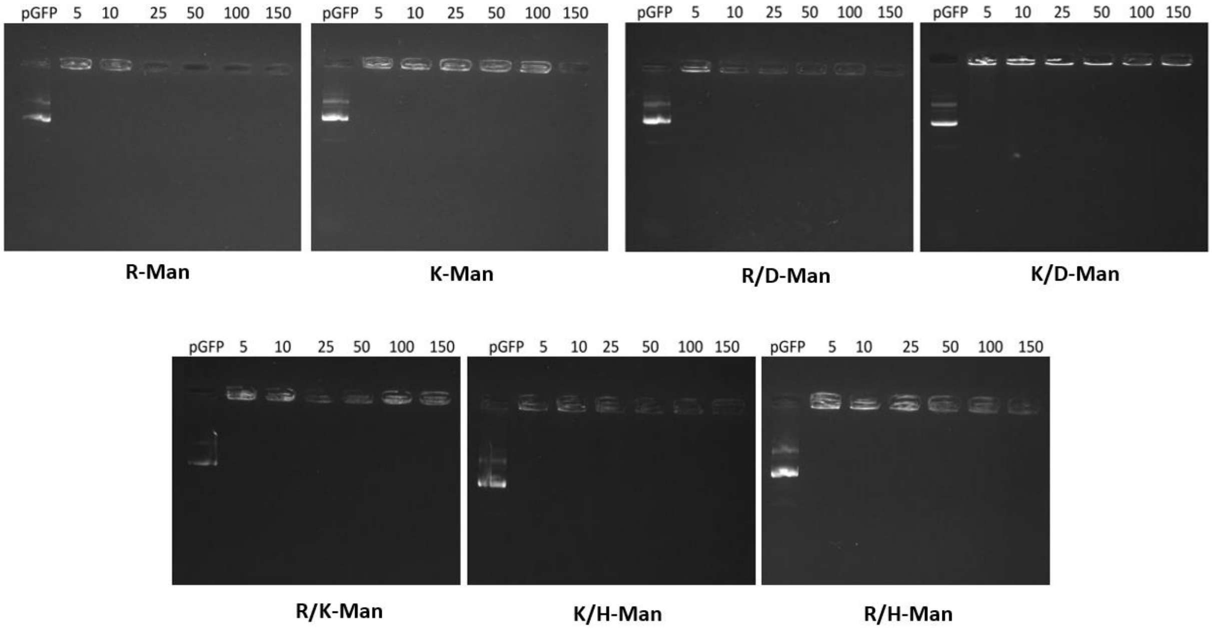


**Figure S2.** Gel retardation assays of polyplexes formulated with distinct mixtures of different OM-C6-PBAEs. Complexes were prepared as described combining cationic and anionic polymers at the established ratios and electrophoresed at 120 V for one hour. Naked pGFP was used as control group in order to evaluate the maximum DNA migration.
